# Characterisation of peripheral blood mononuclear cell microRNA in hepatitis B-related acute-on-chronic liver failure

**DOI:** 10.1038/srep13098

**Published:** 2015-08-12

**Authors:** Wenchao Ding, Jiaojiao Xin, Longyan Jiang, Qian Zhou, Tianzhou Wu, Dongyan Shi, Biaoyang Lin, Lanjuan Li, Jun Li

**Affiliations:** 1 State Key Laboratory for Diagnosis and Treatment of Infectious Diseases, Collaborative Innovation Center for Diagnosis and Treatment of Infectious Diseases, The First Affiliated Hospital, Zhejiang University School of Medicine. 79 Qingchun Rd., Hangzhou, 310003. China; 2 Systems Biology Division, Zhejiang-California International Nanosystems Institute, Zhejiang University.

## Abstract

Hepatitis B-related acute-on-chronic liver failure (HBV-ACLF) is a life-threatening condition and the mechanisms of its development and progression remain unclear. The aim of this study was to define the characteristics of peripheral blood mononuclear cell microRNAs in patients with HBV-ACLF. In this study, novel microRNA (miRNA) biomarkers of peripheral blood mononuclear cells (PBMCs) in patients with HBV-ACLF were characterised by high-throughput sequencing and validated by quantitative real-time polymerase chain reaction (qRT-PCR). The results showed 78 miRNAs were significantly differentially expressed in patients with HBV-ACLF compared to patients with chronic hepatitis B (CHB) and healthy controls. Among patients with HBV-ACLF, 17 dysregulated miRNAs increased or decreased more than 4-fold, of which eight miRNAs had higher expression levels than median level. qRT-PCR validation demonstrated that six miRNAs (hsa-miR-21-5p, hsa-miR-34c-5p, hsa-miR-143-3p, hsa-miR-143-5p, hsa-miR-374a-3p and hsa-miR-542-3p) may be useful as novel biomarkers for the diagnosis of HBV-ACLF. Five novel miRNAs (L-miR-1~5) were detected and two (L-miR-1 and L-miR-3) were significantly differentially expressed in patients with HBV-ACLF. Conclusions: The miRNA expression profile of PBMCs is altered in patients with HBV-ACLF, and a signature of six miRNAs may be a promising biomarker for HBV-ACLF progression.

Hepatitis B virus (HBV) infection is a serious health issue throughout the world, particularly in the Asia-Pacific region. Chronic HBV infection can lead to serious liver dysfunction and diseases, including chronic hepatitis, cirrhosis, and hepatocellular carcinoma (HCC)^1^. HBV-related acute-on-chronic liver failure (HBV-ACLF) is a specific syndrome involving the acute deterioration of liver function and its subsequent effects on multiple other organs^2^. However, the precise mechanisms of HBV-ACLF pathogenesis and progression remain unclear. The establishment of sensitive and specific biomarkers for diagnosis and prediction of HBV-ACLF progression is critical for identifying patients who are high-risk. MicroRNAs (miRNAs), small endogenous non-coding regulatory RNA molecules with a length of approximately 22 nucleotides, participate in a variety of biological and pathological processes, including cell development and differentiation, apoptosis, stress and immune responses, and even carcinogenesis through the regulation of gene expression^3^,^4^. miRNAs as potential biomarkers have been demonstrated in cancer, cardiovascular disease and neurological disease^4^,^5^,^6^. Recent studies indicated that miRNAs might participate in the pathogenesis of liver injury^7^,^8^ and initially showed the significance of the miRNA expression in peripheral blood mononuclear cells (PBMCs) of HBV-ACLF patients^9^. So characterise the miRNA expression profile may help to understand the progression of HBV-ACLF. We hypothesised that the pathological process of HBV-ACLF would result in the alteration of the miRNA profile of PBMCs. In this study, we investigated the miRNA profile of PBMCs among patients with HBV-ACLF who met the criteria based on CLIF-SOFA scores^10^. Initial screening of dysregulated miRNAs was conducted using next-generation sequencing (NGS), and the selected dysregulated and novel potential miRNAs were confirmed by TaqMan probe-based quantitative real-time polymerase chain reaction (qRT-PCR).

## Results

### Qualitative analysis of miRNAs using next-generation sequencing in PBMCs from patients with HBV-ACLF or chronic hepatitis B and healthy controls

To obtain the miRNA expression profiles of human PBMCs and to investigate the alteration of miRNA expression in patients with HBV-ACLF compared to patients with chronic hepatitis B (CHB) and healthy controls, we performed next-generation sequencing of miRNAs isolated from groups of HBV-ACLF patients, CHB patients and healthy controls; each group had 4 subjects, as detailed in Table 1. We identified more than 4 million reliable reads from the sample with the least reads and more than 12 million reliable reads from the sample with the most reads for human miRNAs registered in miRBase v21. There were 1167 miRNAs detected in at least one sample, whereas only 498 miRNAs were detected in all samples. There were 959, 979 and 969 miRNAs detected in at least one sample of the HBV-ACLF, CHB and healthy groups, respectively. Hsa-miR-21-5p was the most abundant miRNA in all samples, with the percentage of mapped reads ranging from 17.8% to 73.1% of the known miRNAs in each sample, though the second most abundant miRNA varied from 2.1% to 11.1% across all samples (Supplemental Table 1). Hsa-miR-21-5p, hsa-let-7g-5p and hsa-miR-26a-5p were reported as abundant miRNAs in a previous study^11^.

### Detection of differentially expressed miRNAs in patients with HBV-ACLF and initial biomarker screening

**Figure 1:**
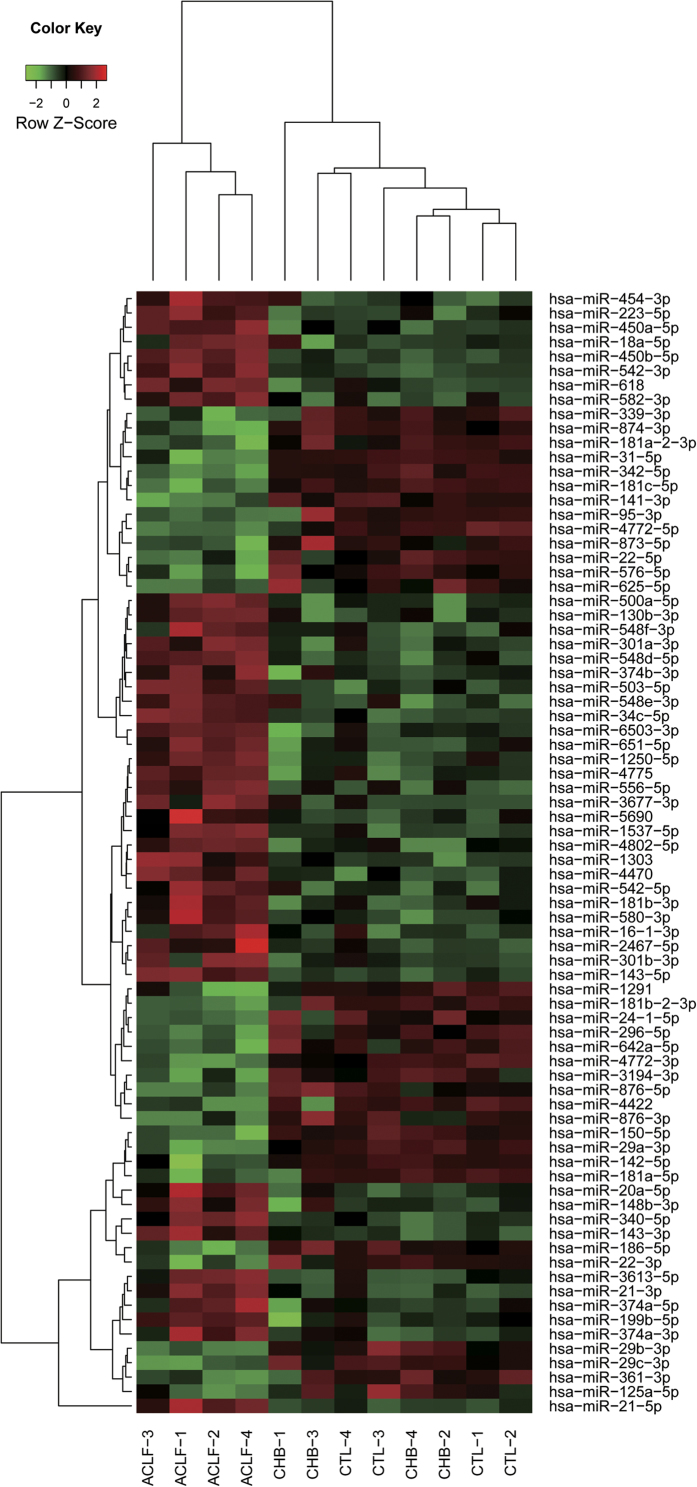
The clustering results of the samples and miRNAs using normalised expression of 78 aberrantly expressed miRNAs in patients with HBV-ACLF. The sample groups are as follows: ACLF, patients with HBV-ACLF; CHB, patients with CHB; CTL, healthy controls.

The analysis of differential expression of miRNAs in patients with HBV-ACLF compared to patients with CHB and healthy controls was performed using DESeq2 with a raw read count of each miRNA. There were 121 miRNAs significantly differentially expressed in patients with HBV-ACLF compared to patients with CHB and 103 differentially expressed miRNAs when HBV-ACLF patients were compared to healthy controls. However, there were no miRNAs that were significantly differentially expressed between patients with CHB and healthy controls. There were 78 common miRNAs in the previous two groups of aberrantly expressed miRNAs, of which 45 were upregulated and 33 were downregulated in patients with HBV-ACLF. The clustering analysis using the normalised expression of these 78 miRNAs showed that all patients with HBV-ACLF were clustered together, which revealed the capacity of miRNAs to distinguish patients with HBV-ACLF from patients with CHB and healthy subjects. The clustering also revealed that hsa-miR-21-5p had distinct differential expression compared with other miRNAs (Fig. 1).

**Figure 2:**
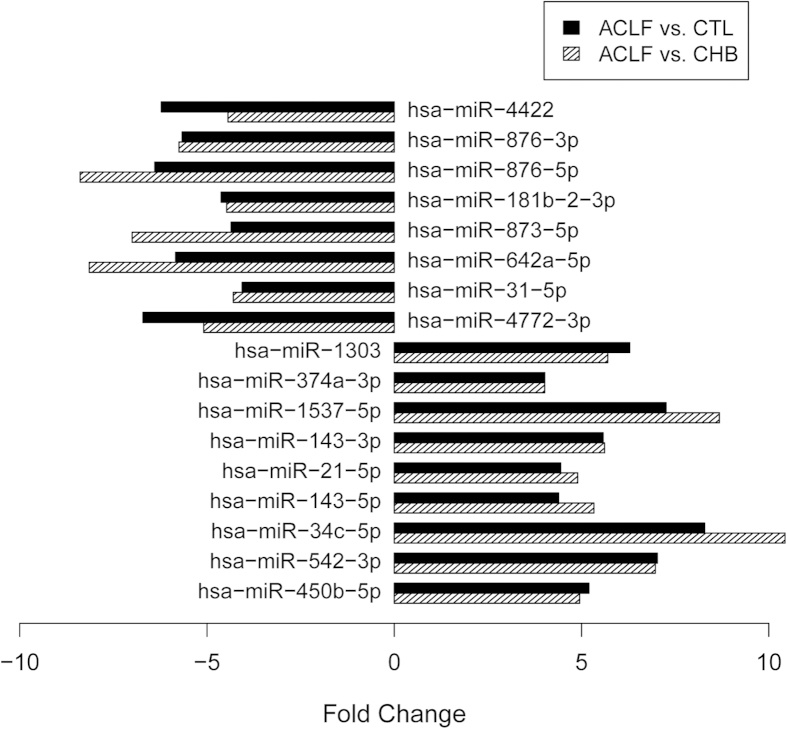
Seventeen miRNAs with more than 4-fold changes in expression levels among patients with HBV-ACLF compared to patients with CHB and healthy controls (CTL).

**Figure 3:**
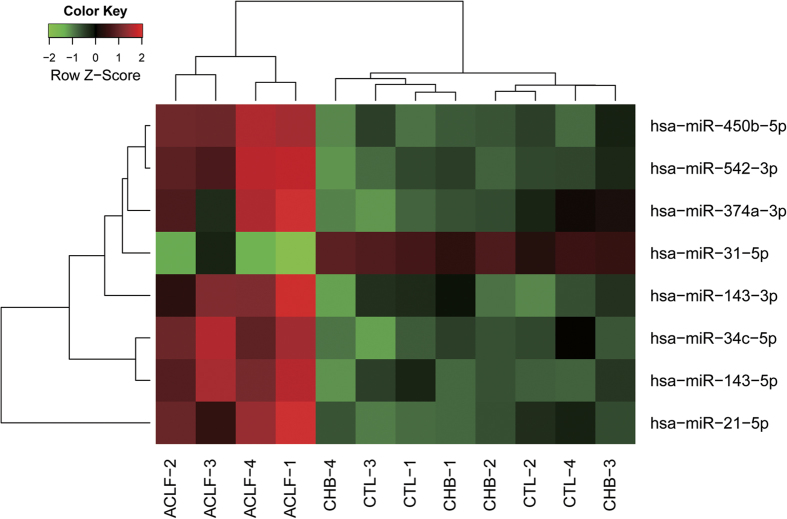
A panel of 8 miRNAs could distinguish patients with HBV-ACLF from patients with CHB and healthy controls. The groups of samples are as follows: ACLF, patients with HBV-ACLF; CHB, patients with CHB; CTL, healthy controls.

To seek appropriate biomarkers for the diagnosis of HBV-ACLF, we chose 17 miRNAs from 78 that were significantly differentially expressed and that had greater than 4-fold expression changes in comparisons of patients with HBV-ACLF to patients with CHB and healthy controls (Fig. 2). Further, from these 17 miRNAs, we selected eight miRNAs with normalised expression levels greater than the median normalised expression level of miRNAs in patients with HBV-ACLF, which included hsa-miR-21-5p, hsa-miR-31-5p, hsa-miR-34c-5p, hsa-miR-143-3p, hsa-miR-143-5p, hsa-miR-374a-3p, hsa-miR-450b-5p and hsa-miR-542-3p. The expression of these eight miRNAs clustered in patients with HBV-ACLF, meaning that these eight miRNAs could potentially be used as a panel of novel biomarkers for HBV-ACLF diagnosis (Fig. 3).

### Validation of the expression levels of eight miRNAs by qRT-PCR

**Figure 4:**
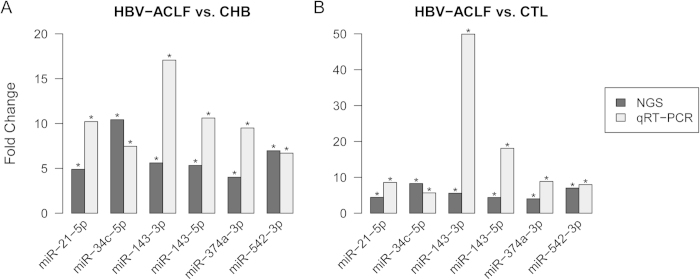
Comparisons of fold changes of the expression results obtained by NGS and qRT-PCR for 6 miRNAs. (**A**) Comparison between HBV-ACLF patients and CHB patients. (**B**) Comparison between HBV-ACLF patients and healthy controls (CTL).

To validate the expression levels of the eight selected miRNAs, we performed qRT-PCR on 90 samples, as detailed in Table 1. According to the results of qRT-PCR validation, we found that the dysregulation of hsa-miR-31-5p and hsa-miR-450b-5p in patients with HBV-ACLF was slight and not significant (data not show), therefore they were excluded in the following analysis. We then compared the fold changes between the initial NGS results and the qRT-PCR validation results for the other six miRNAs (hsa-miR-21-5p, hsa-miR-34c-5p, hsa-miR-143-3p, hsa-miR-143-5p, hsa-miR-374a-3p and hsa-miR-542-3p). When the HBV-ACLF patients were compared to the CHB patients, such six miRNAs were all significantly increased in both approaches. The expression levels of the six significantly differentially expressed miRNAs were increased at least 6.7-fold among HBV-ACLF patients in qRT-PCR validation; the change of hsa-miR-143-3p reached 17-fold (Fig. 4A). When patients with HBV-ACLF were compared to healthy controls, whose levels were similar to the previous results. The expression levels of the six miRNAs were significantly dysregulated also increased at least 5.7-fold in patients with HBV-ACLF, and the change of has-miR-143-3p even reached 49.9-fold (Fig. 4B). The above results indicate a high degree of concordance between the initial screening and the validation study and also indicate that the six miRNAs could potentially be used as a signature for HBV-ACLF.

### Target prediction of differentially expressed miRNAs and GO enrichment analysis

**Figure 5:**
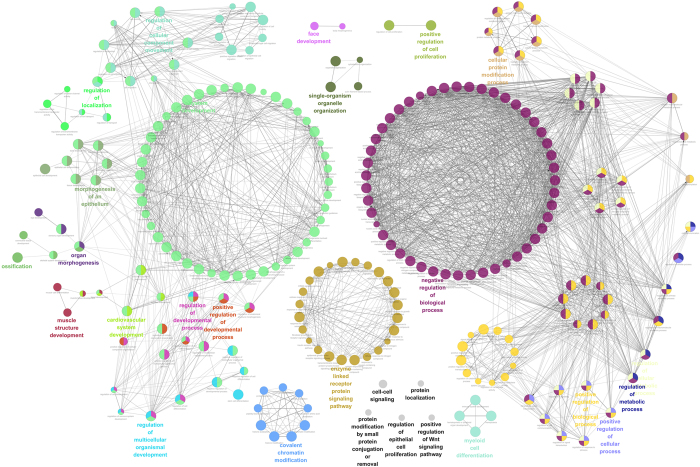
The network of over-represented GO terms. Each node represents a GO term. Each node colour represents a functional group. Terms are connected based on shared genes. The highlight terms are the most significant terms from each group.

To predict the target genes of differentially expressed miRNAs, we searched targets of miRNAs from the miRTarBase and miRDB databases. There were 524 and 1791 targets obtained from the miRTarBase and miRDB databases, respectively, for the six validated dysregulated miRNAs (hsa-miR-21-5p, hsa-miR-34c-5p, hsa-miR-143-3p, hsa-miR-143-5p, hsa-miR-374a-3p and hsa-miR-542-3p). We merged the targets from the two databases and obtained a target set that consisted of 2162 unique targets. The target set was used to perform a GO enrichment analysis. As a result, 256 GO terms were identified and clustered into 23 groups (Fig. 5). We found that the targets of six miRNAs mainly participated in system development, regulation of signalling, regulation of localisation, cellular metabolic process, RNA metabolic process and gene expression.

### Identification and validation of novel miRNAs

**Figure 6:**
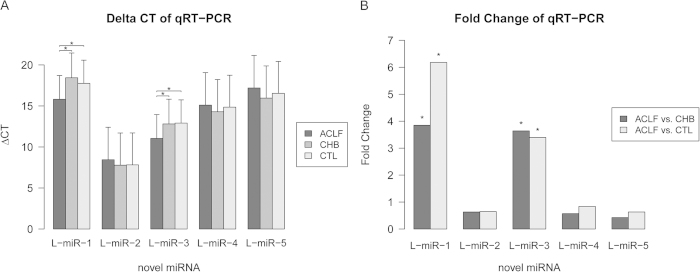
ΔCT values and fold changes of novel miRNAs from qRT-PCR results. (**A**) The ΔCTs of novel miRNAs from qRT-PCR results. (**B**) The fold changes of novel miRNAs in comparisons between HBV-ACLF patients, CHB patients and healthy controls (CTL). (*): The difference is significant (*P* < 0.05).

Initially, 203 novel mature miRNAs were extracted from the results of miRDeep2 according to the selection conditions. After filtering novel miRNAs that aligned to known noncoding RNA (ncRNA) and mRNA sequences, 107 novel mature miRNAs were identified (Supplemental Table 2). We selected the top five abundant novel miRNAs (L-miR-1~5) for validation with qRT-PCR. All five novel miRNAs were detected by qRT-PCR, and their relative expression levels were consistent with the NGS results except for L-miR-1 (Fig. 6A). Furthermore, we found the expression levels of L-miR-1 and L-miR-3 were significantly increased in patients with HBV-ACLF (Fig. 6B), indicating that they might be associated with HBV-ACLF progression.

## Discussion

HBV infection is known to modulate the expression of host cellular miRNAs, which then participate in the development of HBV-related liver diseases, though cellular miRNAs can also regulate HBV gene transcription and replication^12^. Because of the close relationship between HBV reactivation and miRNA regulation, the alteration of miRNA profiles might reflect the progression of liver diseases. Ji *et al.* showed that hsa-miR-122-5p had significantly greater expression among patients with HBV-ACLF than among patients with CHB^13^. This result indicates that the serum miRNA profile can be easily altered by HBV infection. Recently, one study investigated the relationships between the miRNA profile of PBMCs among patients with HBV-ACLF using a microarray, but few of the dysregulated miRNAs were validated via qRT-PCR^9^. Our study is the first to investigate the comprehensive miRNA profile of PBMCs in patients with HBV-ACLF using high-throughput sequencing, and we sought to identify a miRNA-based signature of HBV-ACLF for diagnosis. We identified 78 known PBMC miRNAs that were significantly dysregulated in patients with HBV-ACLF when compared to patients with CHB and healthy controls in the initial screening. To select appropriate biomarkers for the diagnosis of HBV-ACLF, strict criteria were used, including changes in miRNA expression of at least 4-fold and read counts of miRNA greater than the median of the normalised read counts in patients with HBV-ACLF. Under these criteria, seven upregulated miRNAs and one downregulated miRNA were selected for further qRT-PCR validation. Ultimately, the elevated expression levels of six miRNAs (hsa-miR-21-5p, hsa-miR-34c-5p, hsa-miR-143-3p, hsa-miR-143-5p, hsa-miR-374a-3p and hsa-miR-542-3p) were confirmed. In addition, 107 novel miRNA candidates were identified in PBMCs, and five were confirmed with qRT-PCR. Moreover, two novel miRNAs (L-miR-1 and L-miR-3) were significantly upregulated in patients with HBV-ACLF. These results demonstrate that the PBMC miRNA profiles were considerably altered between patients with CHB and those with HBV-ACLF, and the expression patterns of the six-miRNA signature might be able to distinguish patients with HBV-ACLF from patients with CHB.

Hsa-miR-21-5p was the most abundant miRNA in PBMC, as it accounted for an average of 26.4% of the total miRNA population in healthy controls. The results were similar in patients with CHB, whereas the abundance in patients with HBV-ACLF rose to 62.3%. Serum hsa-miR-21-5p was significantly elevated in patients with CHB^14^, though there was no difference in hsa-miR-21-5p in PBMCs from patients with CHB. Additionally, there was no difference compared to healthy controls, which suggests that the PBMC miRNA profile is more stable than the serum miRNA profile in patients with CHB. Previous studies have indicated that hsa-miR-21-5p is involved in inflammatory responses, apoptosis and hepatocyte proliferation and plays roles in liver disease and cancer^15^,^16^,^17^. Because hsa-miR-21-5p is the most abundant miRNA in PBMCs and thus is easily detectable, our study suggests that it could be an appropriate HBV-ACLF diagnostic biomarker. Furthermore, if hsa-miR-21-5p expression is causally related to HBV-ACLF progression, its elevated levels may be associated with disease severity and may therefore be prognostic. Hsa-miR-34c-5p is a member of the has-miR-34 family, which consists of hsa-miR-34a/b/c. Hsa-miR-34c-5p is a tumour suppressor miRNA that is downregulated in numerous cancers^18^,^19^. It has been reported that hsa-miR-34c-5p expression is significantly upregulated in the inflammatory response to damaged cells and has implications for many acute and chronic inflammatory disorders^20^. For this reason, the necrotic cells generated in the process of HBV-ACLF might induce hsa-miR-34c-5p upregulation in PBMCs. Hsa-miR-143-3p and hsa-miR-143-5p are a pair of miRNAs that are generated from the hsa-miR-143 precursor. Both miR-143-5p and miR-143-3p are significantly downregulated in multiple gastric cancer cell lines^21^. The upregulation of hsa-miR-143-3p has been reported to promote cancer cell invasion/migration in HCC and prostate cancer^22^,^23^. Using both miRNAs generated from the same precursor can improve the diagnostic accuracy. Hsa-miR-374a-3p is also a member of a three-miRNA family consisting of hsa-miR-374a/b/c. Few studies have investigated hsa-miR-374a-3p and hsa-miR-542-3p, and this report is the first to characterise the relationship between them and their relationships to liver diseases.

In summary, it is feasible to identify miRNA biomarkers for the diagnosis of HBV-ACLF. Further work in a larger cohort is needed to accurately identify these miRNAs as potential diagnostic markers for HBV-ACLF and to evaluate the significance between ACLF and acute liver failure, as well as to determine if these six highly expressed miRNAs could be applied in other causes of liver failure (i.e., HCV and alcohol and drug use). Meanwhile, different HBV genotype and natural history of HBV reactivations whether affect the progression and miRNA expression profile of patients with CHB also need to be clarified in future study. These six highly expressed miRNAs have the potential to be used as novel biomarkers of the presenting progression of HBV-ACLF, and our results indicated that the pathological process of HBV-ACLF is associated with detectable miRNA characteristics.

## Methods

### Patient information

The study protocol was approved by the Clinical Research Ethics Committee of the First Affiliated Hospital, Zhejiang University School of Medicine. Written informed consent was obtained from healthy volunteers and patients or their legal surrogates prior to enrolment. The experiment methods were carried out in accordance with the approved guidelines. Twelve subjects with HBV-ACLF, CHB and healthy control (n = 4 per group) participated in this initial screening of potential novel biomarkers. Another 90 subjects, with 30 in each group, were enrolled for external validation purposes. The definition of HBV-ACLF corresponded to the CLIF-SOFA score through evaluation of liver, kidney, brain and lung function, coagulation and circulation^10^. Cirrhosis was diagnosed based on previous liver biopsy results or a composite of clinical signs and findings from laboratory tests, endoscopy and radiologic imaging. The levels of serum albumin (ALB), alanine aminotransferase (ALT), aspartate aminotransferase (AST), total bilirubin (TB), creatinine (Cr), sodium, the international normalised ratio (INR), HBV DNA and the model for the end-stage liver disease (MELD) score^24^ for each patient with HBV-ACLF were recorded. For patients with HBV-ACLF, time zero was not the time of the pathologically proven diagnosis but the time of hospital admission. All patients received standard medical therapy during hospitalisation, including high caloric nutrition, lactulose for hepatic encephalopathy, renal replacement for hepatorenal syndrome and uraemic symptoms, and prophylactic antibiotics (quinolones) for the primary prevention of spontaneous bacterial peritonitis^25^,^26^. Patients who were infected with other hepatitis viruses (including A, C, D and E) or who had superimposed viral infections were excluded based on serology. Patients with conditions such as hypertension, diabetes carcinoma, and severe intrinsic renal disease were also excluded. The enrolment criteria for the CHB patients corresponded to the following 2009 American Association for the Study of Liver Disease (AASLD) guidelines^27^: HBsAg^+^ >6 months, serum HBV DNA >10^5^ copies/ml, persistent or intermittent elevation in alanine aminotransferase/aspartate aminotransferase (ALT/AST) levels and liver biopsy showing chronic hepatitis. Thirty-four healthy adults were enrolled as normal controls. The clinical characteristics of the enrolled patients and normal subjects are shown in Table 1.

### RNA extraction, sequencing library preparation and next-generation sequencing

PBMCs were collected using Ficoll-Histopaque (Sigma Aldrich, MO, USA) when the patient was admitted to the hospital prior to treatment. The freshly isolated PBMC samples were suspended in TRIzol reagent (Invitrogen, CA, USA) and immediately performed to extract total RNA. Total RNA extraction of PBMCs was performed using TRIzol reagent following the manufacturer’s instructions and the freshly extracted total RNAs were stored at −80 °C for subsequent testing. Preparation of the sequencing library was performed using the Illumina® TruSeq™ Small RNA Sample Preparation Guide, including steps of adapter ligation, reverse transcription, PCR amplification, and pooled gel purification. The pooled library consisted of sequences with lengths of approximately 150 nt. The library was sequenced using an Illumina HiSeq 2000. The average number of sequencing reads was approximately 10 million.

### Bioinformatics analysis of sequencing data

The sequencing reads were pre-processed by cutting the 3′ adapter sequence using the program cutadapt^28^, and reads that were shorter than 18 nucleotides after clipping were removed. The remaining reads were collapsed to unique reads, and their frequency per sample was recorded. Meanwhile, the singleton reads were removed, which makes the mapping steps more reliable and efficient. After the reads were cleaned, we used the program miRDeep2^29^, which mapped the reads against the human genome (hg19) downloaded from UCSC genome database, mapped the reads against miRNA precursor sequences from miRBase release v21^30^, calculated the read counts of miRNAs for each sample and predicted novel miRNAs. If one read aligned with more than one miRNA, the counts were averagely allocated to each aligned miRNA. At the novel miRNA prediction step, we chose the novel miRNAs with miRDeep2 scores greater than 2 and without rfam alerts. The novel miRNA candidates were further mapped against various known ncRNA sequences (miRNA v21, Rfam v11, RepBase v19.04, NONCODE v3.0 and ncRNA database from Ensembl) with bowtie^31^, and all novel miRNAs aligned to any of these ncRNA sequences (allowing 1 mismatch) were excluded. The novel miRNA candidates that aligned with mRNA sequences from the UCSC genome database were also excluded.

### Prediction and enrichment analysis of the target genes of miRNAs

The target genes of the known miRNAs were obtained from the miRTarBase^32^ and miRDB databases^33^,^34^. The miRNA-target interactions (MTIs) of the miRTarBase database are experimentally validated, whereas the MTIs of the miRDB database are predicted based on support vector machines and high-throughput training datasets. The gene ontology (GO) enrichment analysis of the targets was performed using ClueGO^35^ in Cytoscape^36^. The *P-*values for the biological categories were adjusted with the Bonferroni step-down method and were considered significant if *P* < 0.01.

### Quantitative real time-PCR (qRT-PCR)

For validation purposes, we analysed the expression of miRNAs using qRT-PCR in additional samples. Each group of participants with HBV-ACLF and CHB and the healthy controls had 30 samples. The qRT-PCR was performed with a two-step protocol that required only reverse transcription with a miRNA-specific primer followed by real-time PCR with TaqMan® probes (Invitrogen, CA, USA) as per the manufacturer’s instructions. We selected significantly differentially expressed miRNAs with read counts greater than the median of the HBV-ACLF samples and the most abundant novel miRNAs from all samples for qRT-PCR validation. We used the small nuclear RNA RNU6 as an endogenous control.

### Statistical analyses

For the identification of differentially expressed miRNAs in the HBV-ACLF samples, we first excluded miRNAs with mean read counts <10 in all three groups, as these miRNAs were considered to be uncommon. We then used the DESeq2 package^37^ to analyse the differential expression of miRNAs between patients with HBV-ACLF or CHB patients and healthy controls. The DESeq2 provides methods to test for differential expression based on negative binomial generalised linear models. After DESeq2 analysis, we were able to identify miRNAs with differential expression between two groups, along with logarithmic fold changes and *P* values. Unsupervised hierarchical clustering of the samples from different groups with significantly differential expression of miRNAs was performed using the ‘heatmap.2’ function in the gplots package. The analysis of differential expression in qRT-PCR validation was performed with Student’s t-test. The statistical analyses were performed using the R programming tool. A *P*-value < 0.05 was considered statistically significant.

## Additional Information

**How to cite this article**: Ding, W. *et al.* Characterisation of peripheral blood mononuclear cell microRNA in hepatitis B-related acute-on-chronic liver failure. *Sci. Rep.* **5**, 13098; doi: 10.1038/srep13098 (2015).

## Supplementary Material

Supplementary Information

## Figures and Tables

**Table 1:** The demographic and clinical characteristics of the enrolled patients and healthy subjects.

Clinical parameter	Sequencing group (n = 4 per group)			Validation group (n = 30 per group)		
	HBV-ACLF	CHB	Healthy	HBV-ACLF	CHB	Healthy
Age (years)	33.8 ± 10.2	41.8 ± 8.3	30.8 ± 7.6	45.6 ± 10.1	38.8 ± 11.1	28.4 ± 4.3
Sex (M/F)	4/0	4/0	1/3	24/6	21/9	16/14
ALB (g/L)	33.1 ± 3.6	47.3 ± 2.9	48.7 ± 3.8	30.5 ± 3.8	48.9 ± 2.0	47.7 ± 2.3
ALT (U/L)	489.5 ± 125.7	28.5 ± 5.9	12.5 ± 3.7	386.8 ± 294.0	30.6 ± 19.2	13.6 ± 8.2
AST (U/L)	412.3 ± 20.8	23.3 ± 2.1	16.8 ± 2.6	311.7 ± 229.5	29.9 ± 24.4	16.8 ± 3.1
TB (μmol/L)	568.0 ± 92.6	12.3 ± 4.3	9.8 ± 1.7	363.7 ± 110.9	16.4 ± 6.5	15.5 ± 14.3
Cr (μmol/L)	85.3 ± 23.5	67.3 ± 18.8	62.8 ± 24.2	70.1 ± 20.1	66.9 ± 12.3	63.2 ± 17.2
Sodium (μmol/L)	133.5 ± 2.4	141.5 ± 1.3	139.8 ± 1.0	136.4 ± 4.0	141.2 ± 1.8	139.8 ± 1.1
INR	2.22 ± 0.27	1.10 ± 0.15	NA	2.07 ± 0.37	0.97 ± 0.05	NA
HBV DNA log10(IU/ml)	6.8 ± 7.0	7.1 ± 7.2	NA	7.8 ± 8.5	8.0 ± 8.2	NA
MELD	27.0 ± 2.4	NA	NA	26.1 ± 2.5	NA	NA
