# Supplementary material for: Characterisation of peripheral blood mononuclear cell microRNA in hepatitis B-related acute-on-chronic liver failure
Source: Sci Rep. 2015 Aug 12;5:13098. doi: 10.1038/srep13098 (PMC4533317; doi:10.1038/srep13098)
Supplement: Supplementary Information [file srep13098-s1.pdf]

Characterisation of peripheral blood mononuclear cell microRNA in hepatitis B-related acute-on-chronic liver failure

Wenchao Ding[1,2], Jiaojiao Xin[1], Longyan Jiang[1], Qian Zhou[1], Tianzhou Wu[1], Dongyan Shi[1], Biaoyang Lin[1,2], Lanjuan Li[1], Jun Li[1*]

Contents

Supplemental Table 1.

Supplemental Table 2.

Supplemental Table 1. Summary of miRNAs in each sample

Sample	Known miRNA number	Read count of miRNAs	Percentage of hsa-miR-21-5p (%)	Second abundant miRNA	Percentage (%)
HBV-ACLF-1	721	12,397,041	73.1	hsa-miR-148b-3p	2.1
HBV-ACLF-2	715	8,441,948	63.2	hsa-miR-30e-5p	2.3
HBV-ACLF-3	813	7,035,618	46.9	hsa-let-7g-5p	5.0
HBV-ACLF-4	659	6,448,816	66.1	hsa-miR-30e-5p	2.5
CHB-1	793	4,775,333	24.4	hsa-miR-451a	5.9
CHB-2	808	7,487,423	26.5	hsa-miR-26a-5p	7.5
CHB-3	684	4,374,219	21.1	hsa-let-7g-5p	11.1
CHB-4	754	5,679,992	24.6	hsa-let-7g-5p	7.1
CTL-1	779	6,381,043	22.8	hsa-miR-26a-5p	7.7
CTL-2	757	6,668,115	30.6	hsa-let-7g-5p	7.6
CTL-3	756	5,356,852	17.8	hsa-miR-26a-5p	7.0
CTL-4	786	8,090,160	34.2	hsa-miR-26a-5p	6.2

Supplemental Table 2. Novel miRNA candidates predicted by miRDeep2.

Name	Read count	Mature sequence	Precursor coordinate
L-miR-1	1131	uccacauguaaaaaaaugaauc	chr1:63966763..63966827:-
L-miR-2	1125	ucugccaacccuugucccag	chr11:68946743..68946826:-
L-miR-3	705	ugaggccgagaaggcaaccgcga	chr1:150207085..150207139:+
L-miR-4	249	ucugccaaccuuuguacca	chr8:53115934..53115999:+
L-miR-5	156	uagagcugaauuacuuuccaga	chr1:167440520..167440586:-
L-miR-6	148	uuugacuugaacucauuccccagg	chr5:176932110..176932198:-
L-miR-7	96	ugggcuucucucuggccaca	chr2:85109656..85109712:+
L-miR-8	90	uuauccuccaguagacuaggga	chr8:99405894..99405953:-
L-miR-9	89	gaccucgccgucccgcccgcc	chrUn_gl000220:150005..150061:+
L-miR-10	84	uuggggcuguucucucucccacu	chr1:112783906..112783968:-
L-miR-11	75	uuaguggcucccucugccugca	chr19:57950479..57950542:+
L-miR-12	74	aaccccagacugggaggaccu	chr9:135878152..135878209:-
L-miR-13	66	ugcccggauuaucacuuacuag	chr3:143136938..143136992:-
L-miR-14	60	agcaccugccgagcacugaga	chr17:75675458..75675512:+
L-miR-15	57	caacaggccuugcucugcucacaga	chr3:52557372..52557444:+
L-miR-16	54	acccucaguccguauuggucucu	chr17:7210149..7210207:-
L-miR-17	45	uaugugccuaguggcugcugucu	chr13:27259471..27259537:+
L-miR-18	45	aauccucacuuugaauccaugu	chr2:16612738..16612800:-
L-miR-19	39	uaucugcuguuguccccucagg	chr19:7533657..7533712:+
L-miR-20	39	ugacuacuuuguuugauuuugu	chr6:142308571..142308637:-
L-miR-21	37	uggcuggcugcuccgggcacu	chr10:43966686..43966748:+
L-miR-22	36	acuccucauuuguaaacucagg	chrX:128924417..128924477:+
L-miR-23	33	aagaguuacuagaacuauuca	chr12:21671603..21671661:+
L-miR-24	33	uaggccauuuuggaagcuguuu	chr2:33498399..33498463:+
L-miR-25	32	ggcuccaccuuccuagguuggc	chr22:26968292..26968349:-
L-miR-26	30	agccucccagucuggccugagu	chr1:55784365..55784418:+
L-miR-27	30	caguucaaugguguucagcaga	chr2:74766442..74766500:-

L-miR-28	30	ccuucucgagccuugagugugc	chr3:52427970..52428031:+
L-miR-29	30	uaggacguaugcuuacccuguc	chr7:139422461..139422510:-
L-miR-30	29	agauggggagaacucaauccu	chr4:48222138..48222200:-
L-miR-31	28	agcauaaacugcaugccugcac	chr4:76895032..76895095:-
L-miR-32	27	aaugauggcaucaaaccaggugc	chr19:16477970..16478030:+
L-miR-33	27	cucaccccuguggcacucugggc	chr22:50757156..50757234:+
L-miR-34	27	auuaucucacuuaauacucaac	chr7:18434903..18434962:+
L-miR-35	26	uagacaaucuguguagagugcu	chr11:18026255..18026317:-
L-miR-36	26	auuccuuuuucucuucccucaga	chr11:122928626..122928692:-
L-miR-37	25	uuuauuucaaaggacagcugga	chr2:230195438..230195491:-
L-miR-38	25	aggcgggaauuccaucucauug	chr22:40706263..40706326:+
L-miR-39	24	ccuguucguaaauguuagugga	chr1:193126397..193126457:+
L-miR-40	24	acaugugucugugucugcccagc	chr16:3598217..3598270:-
L-miR-41	24	gcagccgggaaagaacuguc	chr3:194304530..194304584:-
L-miR-42	23	agcccaaagcacuuggcugcccugc	chr16:29816043..29816107:+
L-miR-43	23	uuacaaaaugauuacuguuuacu	chr6:836156..836216:+
L-miR-44	22	uugaacuugggacuaagccugu	chr2:172265618..172265688:-
L-miR-45	21	aauugaacugcucuaugcuacu	chr5:142306557..142306627:+
L-miR-46	20	augugaaauggaauauagaa	chr2:172605840..172605902:+
L-miR-47	20	ugaacuugacuuacagggugaa	chr5:94251400..94251463:-
L-miR-48	20	aauuuuaagggauaacag	chrX:3450117..3450177:-
L-miR-49	19	agaaucuguugguaaagccucu	chr1:221987294..221987355:-
L-miR-50	19	guccccagacucucccgggcugu	chr2:17934536..17934597:-
L-miR-51	19	uuuuuugcuggaacauuucuggu	chr6:39042089..39042159:+
L-miR-52	19	uaaacacuggccaccagcagu	chr9:95798465..95798518:+
L-miR-53	19	uggaaaauuccugggaagucu	chr9:28832561..28832614:-
L-miR-54	18	ugcgugaguccgggcucccagu	chr15:63779648..63779720:-
L-miR-55	18	gaagcagcgccugucgcaacucg	chr17:76136837..76136896:+
L-miR-56	18	caaaugagaacccagauaucug	chr20:12440539..12440606:+

L-miR-57	18	uucuuggaccuugcuucagacc	chr5:171560962..171561020:-
L-miR-58	17	aaaugaaucauguugggccugu	chr10:115051389..115051439:-
L-miR-59	17	ugccuugcccucucccucuagg	chr3:48717661..48717717:-
L-miR-60	16	uugccucucccgucccuguagu	chr19:3201463..3201522:+
L-miR-61	16	aaacucugagcaagaacuccccu	chr21:36824270..36824337:+
L-miR-62	16	uauacugaacagugcacccga	chr22:47167319..47167372:+
L-miR-63	16	ucuucaggaacucuggcuaacu	chr9:37804227..37804283:+
L-miR-64	15	uuuggccuuuuauguucuuga	chr1:215761740..215761807:+
L-miR-65	15	caacguacuaagaauaaauuuc	chr12:29416584..29416644:+
L-miR-66	15	ugagcuagggauaucuccugaga	chr4:39240137..39240200:+
L-miR-67	15	ugauuguuuguggaauaaauga	chr9:98645113..98645170:+
L-miR-68	15	uacauugaauuuguggauuacu	chrY:15405891..15405953:-
L-miR-69	14	aaacuuaugaaaagugacuguuu	chr10:4926783..4926851:+
L-miR-70	14	aagagcucucuggcuuugccu	chr10:103986836..103986892:+
L-miR-71	14	uauuacguuauacaguauauacu	chr9:117692175..117692239:+
L-miR-72	13	uuaguucuguggcaacaucgau	chr1:178712691..178712748:+
L-miR-73	13	caaacaggccuagcacaguga	chr11:92933053..92933118:+
L-miR-74	13	uauauauguuguguauguauacg	chr11:44254690..44254755:+
L-miR-75	13	ccuuccccacccucuccugcagc	chr19:13063444..13063504:+
L-miR-76	13	ucaccucucaaacuuccucaga	chr19:35500733..35500793:+
L-miR-77	12	ucuguccugggacgcuuggugucc	chr20:47337764..47337827:-
L-miR-78	12	uuugcagaauggugcuugugcu	chr4:15719466..15719522:+
L-miR-79	12	uaggagaacauaagggaucaau	chr6:24819039..24819096:+
L-miR-80	12	uaggagaacauaagggaucaau	chr6:24819036..24819093:-
L-miR-81	11	ucuggcuccuuucuaaucacu	chr12:81300351..81300410:-
L-miR-82	11	uuccccccaaaauucauaauguu	chr13:35537587..35537649:+
L-miR-83	11	uauauauaaaaugccuguuaaga	chr15:65553703..65553763:-
L-miR-84	10	acacucaucaacugugccugac	chr1:147829303..147829365:+
L-miR-85	10	acacucaucaacugugccugac	chr1:147803763..147803824:-

L-miR-86	10	aaacaacuuguuucucuugcuu	chr12:62609452..62609507:+
L-miR-87	9	ugugggcgacaagguuugcugc	chr22:24833922..24833985:+
L-miR-88	9	agcauaaacugcaugccugcac	chr4:76895073..76895126:-
L-miR-89	8	uuaauauguacugacaaagcgu	chr10:5729070..5729133:+
L-miR-90	8	ugagagcucguguagaaagcc	chr12:11963194..11963255:+
L-miR-91	8	cuacugucacucucucguc	chr19:15575194..15575248:-
L-miR-92	7	aauagugucuagaauaucuug	chr2:136092844..136092898:-
L-miR-93	6	agcuccugacauugucaccuga	chr2:97787824..97787878:+
L-miR-94	6	agcuccugacauugucaccuga	chr2:96648852..96648906:-
L-miR-95	6	agcuccugacauugucaccuga	chr2:98197775..98197829:-
L-miR-96	6	uccuuucagaugguucucuaga	chr2:218905712..218905767:-
L-miR-97	5	acucuuccaaugugcacuuaga	chr1:168490312..168490372:+
L-miR-98	5	uaucagauauucuggauuacuca	chr15:49622626..49622685:+
L-miR-99	5	ugaaagaugacacguguugacu	chr2:198159017..198159099:-
L-miR-100	4	uuaauuaagcagaguagugacu	chr1:162205411..162205475:-
L-miR-101	4	uuaauuaagcagaguagugacu	chr1:162205414..162205478:+
L-miR-102	4	aaucaucuaugaacacugaagu	chr2:85935693..85935749:-
L-miR-103	3	agcccagcccucuucugucucc	chr1:200960858..200960911:-
L-miR-104	3	aagauauucuagacacuauuc	chr2:136092846..136092898:+
L-miR-105	3	uaaucacuagagcggggacuca	chrX:129202337..129202396:-
L-miR-106	2	uuuggggauucuaagaggaag	chr17:42344332..42344389:+
L-miR-107	1	uauauauaaaaugccuguuaa	chr15:65553708..65553764:+
